# Analysis of risk factors associated with cervical HPV infection and their effects on female sexual function and anxiety: a multicenter cross-sectional study based on Chinese women

**DOI:** 10.3389/fonc.2024.1468160

**Published:** 2024-10-24

**Authors:** Haoye Wang, Keyi Nie, Zixuan Liu, Yumeng Zhao, Yingxin Ha, Huanyan Zhang, Dongwei Mao

**Affiliations:** ^1^ Shenzhen Hospital of Guangzhou University of Chinese Medicine (Futian), Shenzhen, China; ^2^ Department of Gynecology, Heilongjiang University of Traditional Chinese Medicine, Heilongjiang, China; ^3^ Department of Brain Disease, The First Affiliated Hospital of Shaanxi University of Traditional Chinese Medicine, Shaanxi, China; ^4^ Department of Gynaecology, The First Affiliated Hospital of Shaanxi University of Traditional Chinese Medicine, Shaanxi, China

**Keywords:** cervical HPV infection, anxiety, sexual function, cervical cancer, cross-sectional studies

## Abstract

**Introduction:**

This research aimed to explore the determinants of cervical HPV infection and evaluate how cervical cancer screening outcomes influence sexual functioning and anxiety among women across three provinces in China.

**Methods:**

Study participants were categorized into HPV-positive or HPV-negative groups, after which they completed the General Characteristics Questionnaire and the SAS Anxiety Scale. The HPV-positive cohort was further divided into four subgroups: Subgroup 1 consisted of individuals with HPV types 16 or 18 but no cytological abnormalities. Subgroup 2 included those with cytological abnormalities and infections with HPV types 16 or 18. Subgroup 3 included participants infected with high-risk HPV types other than 16 and 18, who did not show cytological abnormalities, while Subgroup 4 encompassed individuals with both cytological abnormalities and infections with high-risk HPV types other than 16 and 18. All participants were assessed using the Female Sexual Function Index Scale (FSFI), which measures sexual function across these subgroups.

**Results:**

A total of 531 questionnaires were analyzed. Logistic regression identified several risk factors for HPV infection, including manual labor, service occupations, other occupations, low- and middle-income groups, and cervical columnar epithelial ectasia, as well as an increase in the number of previous sexual partners. Conversely, protective factors included condom use during sexual activity and mutual genital hygiene prior to intercourse. The incidence of lower genital tract infections was significantly higher in the HPV-positive group compared to the HPV-negative group, with rates of chlamydia (13.3% versus 4.6%, P<0.001), gonococcal infections (5.2% versus 0.4%, P<0.001), and mycobacterial diseases (38.9% versus 23.4%, P<0.001). Additionally, anxiety levels were significantly higher in the HPVpositive group compared to the HPV-negative group (p<0.005). Anxiety levels and cytological outcomes were significantly correlated across the four HPV-positive subgroups (p<0.05), with type 16/18 infections being associated with notably lower FSFI scores compared to other high-risk HPV types (p<0.05).

**Conclusions:**

The findings suggest that infections with HPV type 16/18, especially when accompanied by abnormal cytological findings,significantly elevate anxiety and distress in women and are associated with reduced FSFI scores. These results highlight the complex impact of HPV infection on women’s sexual health and psychological well-being with HPV type 16/.

## 1 Introduction

Cervical cancer ranks as the fourth most common cancer globally, presenting significant health challenges for women worldwide. In regions with limited resources, particularly those with a human development index below 0.80, cervical cancer poses an even greater threat. A striking 84% of cervical cancer cases and 88% of related deaths occur in these areas. Notably, China has the highest incidence, with 106,000 cases, where the substantial burden of the disease can be largely attributed to inadequate or poor-quality health data (1).This situation highlights the urgent need to expand HPV vaccination and screening programs in these regions to reduce the impact of the disease. Projections suggest a concerning trend: by 2040, the number of new cervical cancer cases is expected to rise to 798,000, while fatalities are anticipated to increase to 481,000. These figures represent a 32% rise in incidence and a 40.8% rise in mortality rates compared to 2020, stressing the importance of comprehensive studies on HPV infections across various Chinese provinces (2). The link between cervical cancer and infection with human papillomavirus (HPV) has been well-established, with 80% to 99% of cervical cancer cases associated with HPV. Out of the 448 identified HPV types, health authorities have classified 12 as class 1 carcinogens due to their high oncogenic potential. Other risk factors contributing to the development of cervical cancer include sexually transmitted diseases such as HIV and Chlamydia trachomatis. Lifestyle choices, such as tobacco use and a history of multiple abortions, also play a significant role in increasing the risk. These factors contribute to the disparities and burdens of cervical cancer worldwide (3). Most HPV infections do not persist, typically resolving spontaneously within two years. However, for infections involving high-risk HPV types, progression to cervical cancer can take approximately 25–30 years if the infection does not resolve (4). Therefore, comprehensive research into the factors influencing the persistence of infections with high-risk HPV is essential. Identifying these determinants is crucial for developing effective strategies for preventing and treating such infections.

Pathogens such as bacteria, viruses, and fungi are the primary causes of infections in the female reproductive system (5). Key pathogens include Trichomonas, mycobacteria, mycoplasma, and Chlamydia (6). Observational data from a large study in Hunan Province, China, revealed that co-infections involving Chlamydia trachomatis and HPV were present in 25.0% of Chlamydia trachomatis cases and 7.6% of HPV cases (7).These co-infections significantly increase susceptibility to additional infections by either HPV or Chlamydia trachomatis. Such findings highlight the need for stronger preventive measures against genital tract infections and cervical lesions. Moreover, the psychological impact of HPV infections on women is profound. Most women report negative psychological effects, such as anxiety and fear, upon receiving positive HPV test results or a diagnosis of persistent HPV infections. Some women may also experience sexual dysfunction as a result. It has been documented that the mental health of individuals infected with HPV can be as important as, or even more critical than, their physical health (8). Negative societal perceptions, such as associations with promiscuity and infidelity, exacerbate the stigma attached to an HPV-positive diagnosis, further increasing psychological distress among affected women (9). Women infected with high-risk HPV strains are particularly prone to increased despair and emotional turmoil. This psychological distress can significantly reduce their quality of life, with conditions such as depression and anxiety impacting their ability to fully enjoy their sexual relationships (10). Thus, a deeper understanding of the psychological well-being and sexual health of HPV-infected women in China could help develop more effective intervention strategies.

In the context of disease prevention and management, psychosocial interventions are increasingly recognized as essential. This research focuses on the risk factors related to cervical HPV infections and explores how different HPV strains and cytological results affect sexual health and anxiety levels in women. The findings contribute to foundational knowledge necessary for improving psychological interventions and guiding follow-up, diagnostic procedures, and treatment strategies for those infected with HPV. By analyzing the interactions between different HPV types and cervical cytology outcomes, this study aims to refine approaches to managing the psychological aspects of HPV infections, ultimately supporting comprehensive care for patients.

## 2 Methods

### 2.1 Study design

This large-scale, multicenter, population-based cross-sectional study is planned to take place between 2022 and 2024 across diverse geographical regions in China. It involves collaboration among three tertiary hospitals, each selected for its unique regional characteristics. The participating institutions include the First Affiliated Hospital of Shaanxi University of Traditional Chinese Medicine, the Shenzhen Hospital (Futian) of Guangzhou University of Traditional Chinese Medicine, and the First Affiliated Hospital of Heilongjiang University of Traditional Chinese Medicine. The primary objective of this study is to conduct an in-depth analysis of risk factors associated with HPV infection and to explore the impact of HPV diagnosis on anxiety and sexual health. Ethical approval for the study was granted by the Institutional Review Board of the Guangzhou University of Chinese Medicine, based at its Shenzhen Hospital (Futian) branch, under approval number GZYLL(KY)-2024-039. This approval confirms the study’s compliance with ethical standards in medical research.

### 2.2 Study population

**Figure 1:**
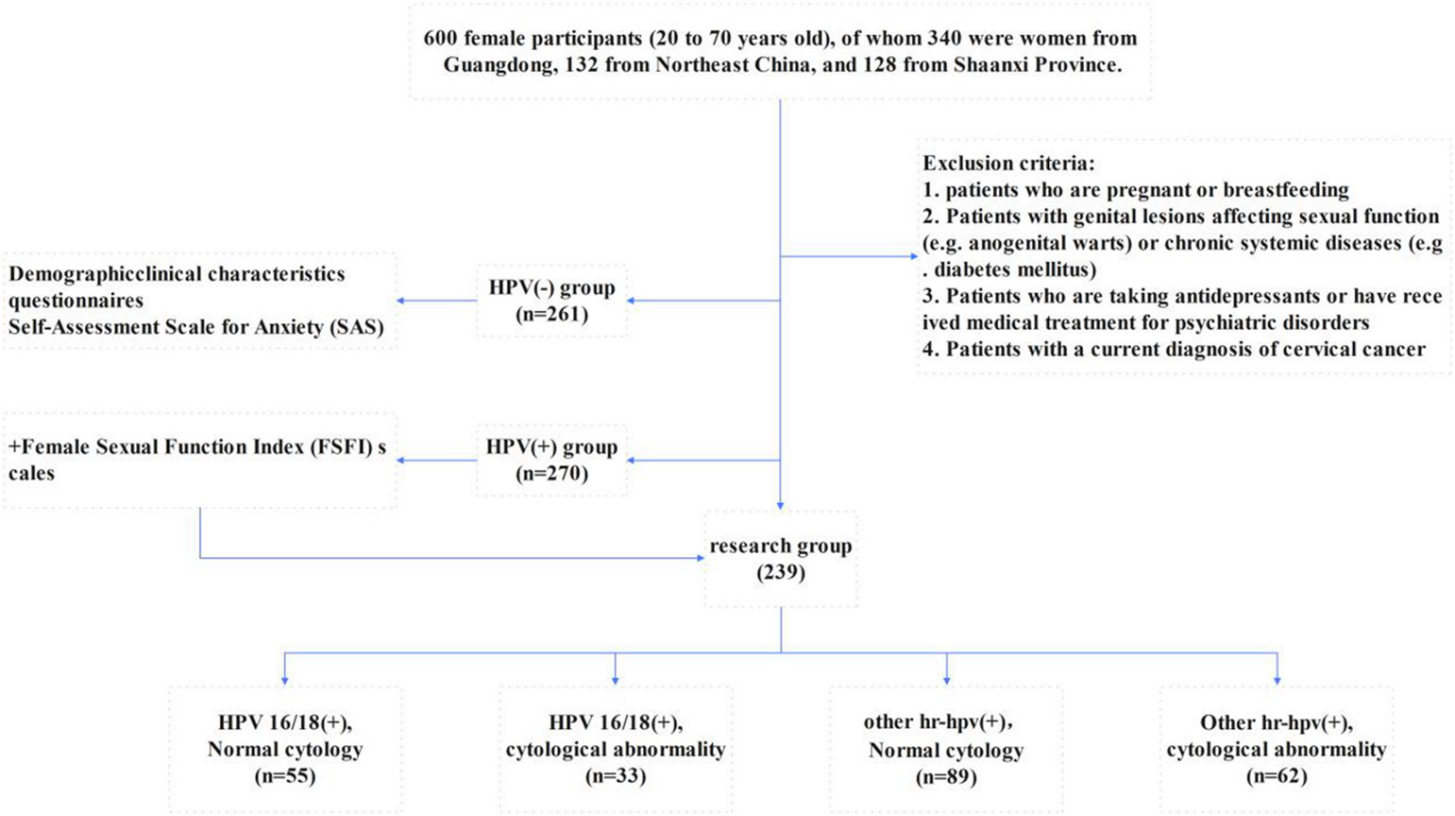
Flowchart of the study.

**Figure 2:**
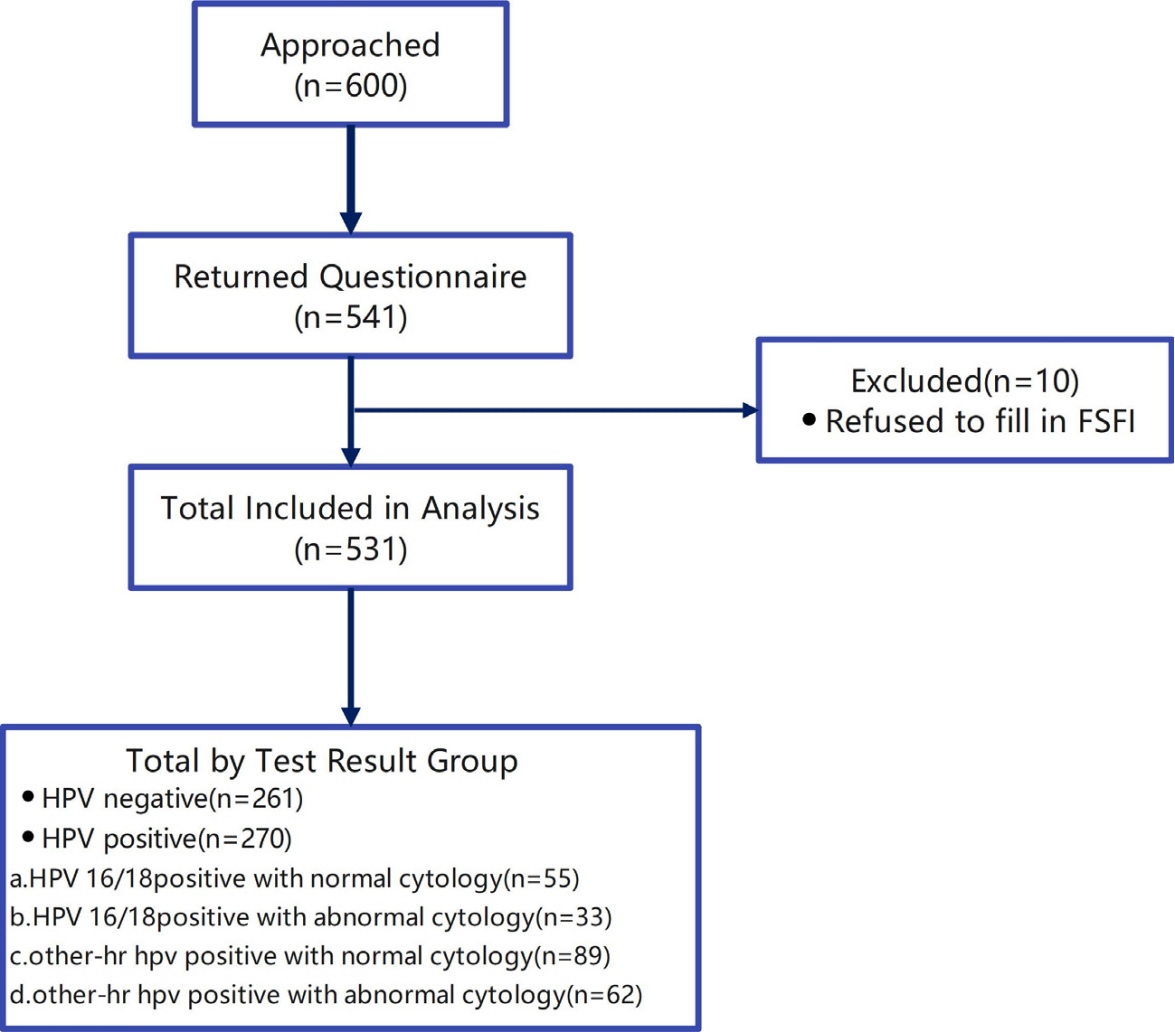
Participant screening process.

As part of a systematic cervical cancer screening program, 531 women aged between 20 and 70 years, who met the study’s inclusion criteria, completed and signed informed consent forms. These participants were then administered a detailed questionnaire at the gynecological clinics of the three selected hospitals. Samples with incomplete questionnaire information were excluded from the study. **Figures 1**, **2** summarize the inclusion and exclusion criteria for each aspect of the study.

### 2.3 Procedure

In this study, 600 women who presented for cervical cancer screening at the gynecological outpatient clinics of three hospitals were selected to undergo HPV genotyping and liquid-based thin-layer cytology. The Polymerase Chain Reaction (PCR), combined with reverse dot hybridization techniques, was used to systematically identify and categorize both high-risk and low-risk human papillomavirus (HPV) types. Specifically, the assay successfully identified a wide range of high-risk HPV types, including 16, 18, 31, 33, 35, 39, 45, 51, 52, 53, 56, 58, 59, 66, 68, 73, 82, and 83. The low-risk HPV types detected through this method were types 6, 11, 42, 43, and 81. The cytological evaluation process categorized samples into two primary groups: those with normal cytological patterns and those with abnormal cytological features. The abnormal category was further subdivided into cases presenting with low-grade squamous intraepithelial lesions (LG-SIL), high-grade squamous intraepithelial lesions (HG-SIL), and atypical squamous cells of undetermined significance (ASC-US) (11). Additionally, the study included further cytological findings such as atypical squamous cells of undetermined significance (ASC-US), low-grade squamous intraepithelial lesions (LSIL), high-grade squamous intraepithelial lesions (HSIL), and atypical glandular cells (AGC). This comprehensive analysis provided a detailed overview of the cytological landscape in HPV-positive individuals. These findings require follow-up procedures such as colposcopy and biopsy to investigate and manage suspicious lesions effectively, thereby improving the diagnostic process and aiding in the subsequent treatment planning for potential cervical abnormalities.

Patients underwent a structured notification process, during which results from HPV testing and cytological analysis were communicated via telephone. They were advised to visit the hospital within one month to collect their detailed test reports and were provided with a questionnaire by the research team during their visit. Researchers assisted those who had difficulties understanding or completing the scales in the questionnaire. Individuals who tested negative for HPV were grouped as controls and were required to complete a questionnaire covering demographic information (such as age, gender, occupational status, marital status, educational background, and annual household income) and clinical characteristics (including previous health conditions, pregnancy and childbirth history, lifestyle habits, and contraceptive use). This group also completed the Self-Assessment Scale for Anxiety (SAS) to evaluate their anxiety levels. Conversely, participants identified as carriers of high-risk human papillomavirus (HR-HPV) were required to provide comprehensive responses to the primary questionnaire set and the Female Sexual Function Index (FSFI). This additional tool is designed to assess various aspects of sexual health, providing a deeper understanding of the impact of HR-HPV on sexual functionality. To ensure the accuracy and completeness of the collected data, researchers reviewed the filled questionnaires immediately, identifying and addressing any omissions or logical inconsistencies. This thorough approach aimed to improve the reliability and validity of the study’s findings.

The classification of the HPV-positive cohort was systematically organized into four distinct groups based on their HPV genotype and cytological results. These groups included: (a) individuals positive for HPV16/18 with normal cytology; (b) individuals positive for HPV16/18 with abnormal cytology; (c) individuals positive for other high-risk HPV types with normal cytology; and (d) individuals positive for other high-risk HPV types with abnormal cytology (12). For all individuals in the first two groups, medical advice was provided to undergo colposcopy, a recommendation shown in **Figure 1**. This stratification facilitated targeted follow-up procedures, improving the management and monitoring of potential cervical health risks associated with different HPV genotypes and cytological findings.

### 2.4 Anxiety Self-Assessment Scale

The Self-Assessment Scale for Anxiety (SAS) evaluates anxiety symptoms experienced over the past week. It consists of 20 items, with a standard threshold score of 50 points. Scores below this threshold suggest a normal psychological state, while scores above 50 indicate the presence of anxiety symptoms, with higher scores reflecting greater symptom severity. Specifically, scores between 50 and 59 indicate mild anxiety, scores between 60 and 69 denote moderate anxiety, and scores of 70 or above classify as severe anxiety. Internal consistency analysis of the scale shows alpha coefficients ranging from 0.864 to 0.955, indicating a high degree of item differentiation. The scale’s structure, including the number of items and factor configurations, was well-balanced, ensuring high overall item quality and minimal measurement errors, thus meeting the essential criteria for psychometric evaluation (13).

### 2.5 Female Sexual Function Index Scale

In a large-scale validation study conducted across multiple centers in China, the effectiveness of the Female Sexual Function Index (FSFI) was rigorously evaluated to determine its suitability as a clinical tool for assessing female sexual dysfunction. The FSFI comprises 19 individual items, organized into six main dimensions: desire, arousal, lubrication, orgasm, satisfaction, and pain. Each dimension is assessed using specific scales: the sections for desire, lubrication, orgasm, and pain utilize a scoring range of 0 to 5, indicating varying levels of severity or frequency. In contrast, the dimensions of satisfaction and arousal are rated on a scale of 1 to 5 to accommodate the nuances of these aspects of sexual function. A higher cumulative FSFI score indicates better sexual function, with a content validity coefficient of 0.953 (14).

### 2.6 Statistical analysis

Data were entered into Excel sheets using a double-checking method to ensure accuracy. All statistical analyses were conducted using SPSS 25.0 statistical software. For the analysis of continuous variables, the data were expressed as mean ± standard deviation, and appropriate tests were selected based on the distribution and variance characteristics. The independent samples t-test was used for data that were normally distributed with homogeneous variance. For data that did not meet these criteria, the rank sum test was applied. Categorical data were presented as ratios or component ratios, and comparisons between groups were made using the chi-square (χ^2^) test. To identify risk factors for HPV infection, differences between categorical variables were analyzed using the chi-square test for epidemiological questionnaire data, while differences between continuous variables were assessed using the rank sum test or ANOVA. Statistically significant variables were further analyzed using one-way regression analysis and multifactor logistic regression analysis. Anxiety was treated as a categorical variable when comparing the HPV-infected and uninfected groups, with differences assessed via the χ^2^ test. Two logistic regression models were developed to explore the correlation between anxiety and HPV infection. In Model I, no adjustments were made for confounding variables. In Model II, adjustments were made for factors such as the number of miscarriages, dietary patterns, and infections with Chlamydia, gonococcus, and mycobacteria. Sexual function scores were treated as continuous variables for within-group comparisons of the study cohort. Given that the distribution of sexual function scores did not meet normality assumptions, the rank sum test was used for analysis. Two models were also developed to assess the correlation between sexual function and the study cohort. In Model I, no adjustments were made for confounding variables, while in Model II, adjustments were made for infections with Chlamydia, gonococcus, Trichomonas vaginalis, mycobacteria, and cervical columnar epithelium. Statistical significance was defined as a p-value of less than 0.05. Values greater than 0.05 were considered not statistically significant, while values less than 0.05 indicated statistically significant differences.

## 3 Results

### 3.1 Distribution of HPV genotypes and prevalence

**Figure 3:**
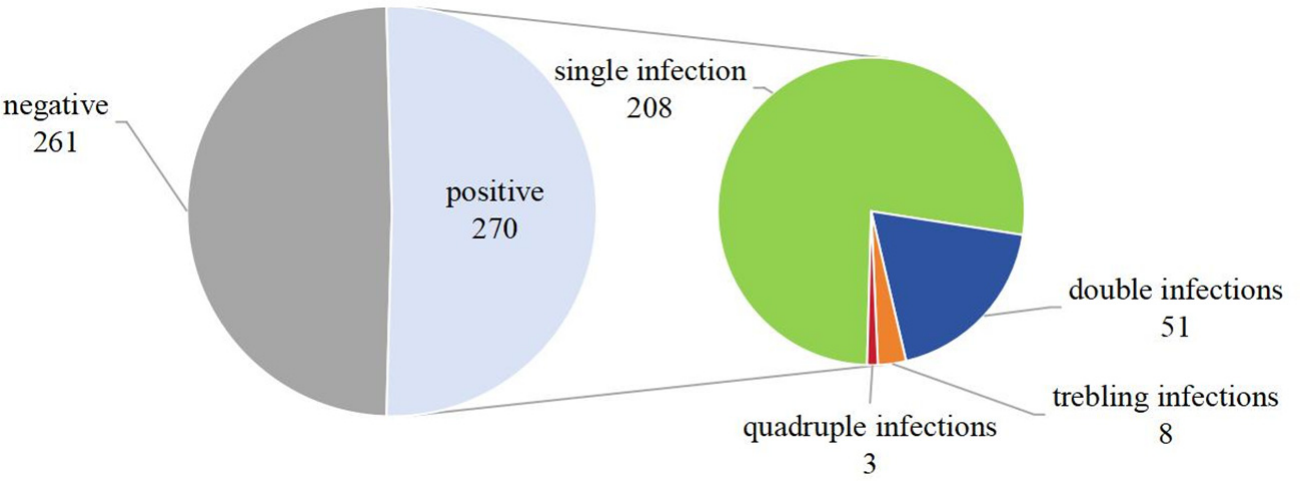
Distribution of HPV infection in 531 patients.

**Figure 4:**
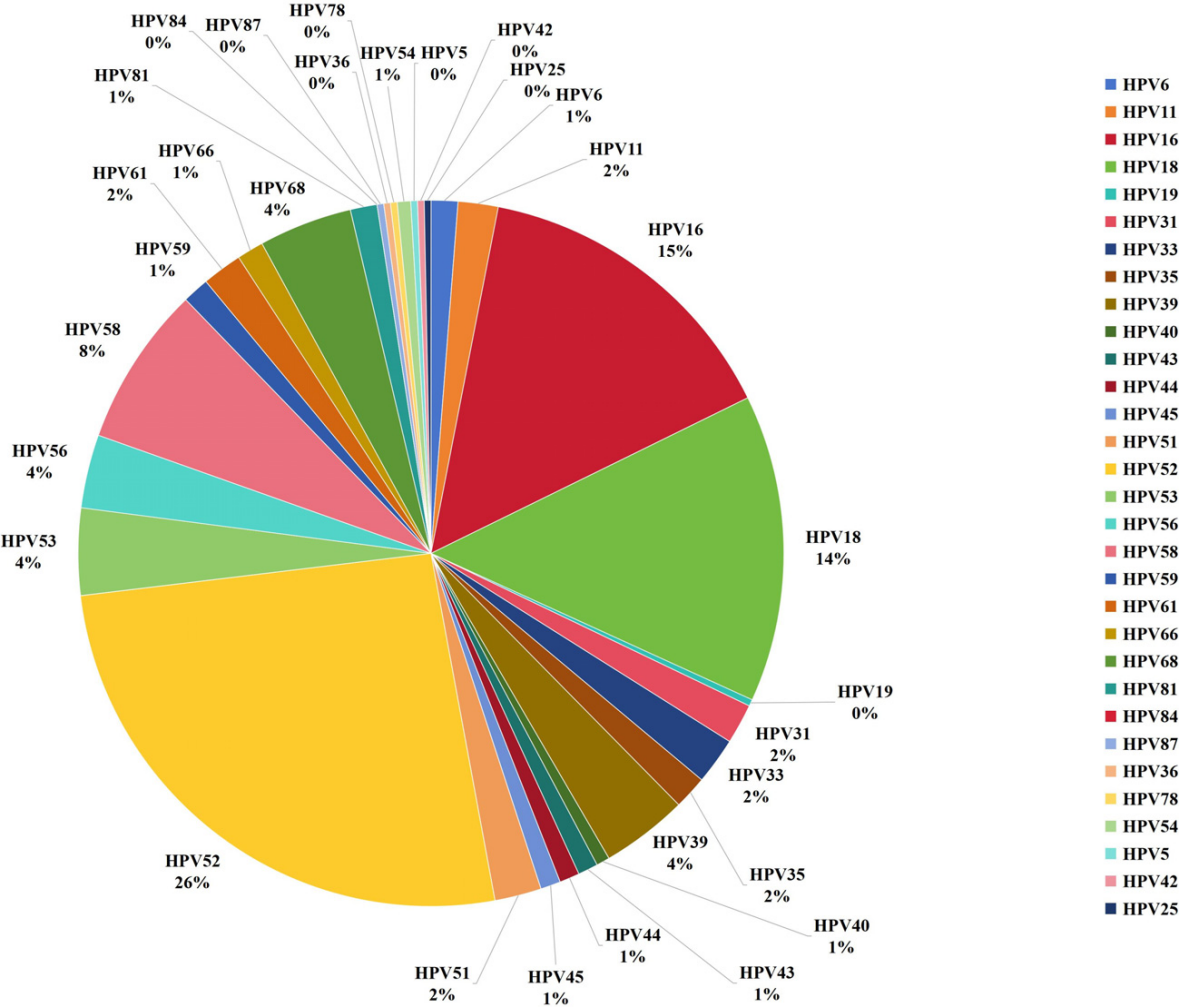
Percentage of human papillomavirus (HPV) infections.

A total of 88% of the women (n=541) returned to the hospital to retrieve their test results and complete the questionnaire, though 10 women chose not to complete the FSFI. As a result, the final sample size analyzed in this study comprised 531 women (as shown in **Figure 2**), with an average age of 39.7 ± 10.2 years. Among these, 270 cases were identified as positive for HPV. The distribution of the positive cases showed that 208 women were infected with a single HPV genotype, representing 77% of the positive cohort; 51 women were infected with two HPV genotypes, accounting for 18.9%; and 11 women had infections involving multiple genotypes (more than two subtypes), making up 4.1%. Further analysis revealed that 239 of the infections were classified as high-risk HPV types, constituting 88.5% of the positive cases. The top five HPV genotypes detected among the positive cases were HPV52, found in 87 cases (32.2% of the positive cohort), followed by HPV16 in 49 cases (18.1%), HPV18 in 48 cases (17.8%), HPV58 in 25 cases (9.3%), and HPV68 in 15 cases (5.6%) (as depicted in **Figures 3**, **4**).

### 3.2 Characteristics of the participants

The statistical analysis of this study comprehensively assessed various demographic characteristics, including age, birthplace, educational background, marital status, total number of pregnancies, childbirths, and smoking status. The findings indicated that there were no significant differences in these demographic parameters between individuals who tested positive or negative for HPV, with p values greater than 0.05 across all comparisons. The data showed that women who began sexual activity at a younger age, had undergone more frequent abortions, and had two or more sexual partners exhibited higher rates of HPV infection. Among the reported contraceptive methods, condom use was the most common (60.1%), followed by intrauterine devices (IUDs) at 17.1%, other methods at 9.8%, and 13% of participants reported not using any form of contraception. The specific characteristics are detailed in **Table 1**.

**Table 1:** Basic characteristics of the study population (n=531).

Characteristic	Total (n=531)	HPV(-) (n=261)	HPV(+) (n=270)	χ2	P
Age, years (n,%)				4.515	0.341
20-29	95 (17.9)	44 (16.9)	51 (18.9)		
30-39	193 (36.3)	89 (34.1)	104 (38.5)		
40-49	137 (25.8)	75 (28.7)	62 (23)		
50-59	89 (16.8)	47 (18)	42 (15.6)		
60-69	17 (3.2)	6 (2.3)	11 (4.1)		
Birthplace (n,%)				0.288	0.591
municipalities	285 (53.7)	137 (52.5)	148 (54.8)		
countryside	246 (46.3)	124 (47.5)	122 (45.2)		
Education Level (n,%)				4.878	0.087
Primary or Junior school	79 (14.9)	44 (16.9)	35 (13)		
High school	163 (30.7)	69 (26.4)	94 (34.8)		
Undergraduate and above	289 (54.4)	148 (56.7)	141 (52.2)		
Profession (n,%)				18.995	<0.001
Freelance or housewife	151 (28.4)	86 (33)	65 (24.1)		
Manual labor or services	128 (24.1)	52 (19.9)	76 (28.1)		
Institutional or specialized technical staff	98 (18.5)	61 (23.4)	37 (13.7)		
Others	154 (29)	62 (23.8)	92 (34.1)		
household income (n,%)				13.824	0.003
≤3000	63 (11.9)	39 (14.9)	24 (8.9)		
3000-5999	159 (29.9)	60 (23)	99 (36.7)		
6000-8999	127 (23.9)	68 (26.1)	59 (21.9)		
≥9000	182 (34.3)	94 (36)	88 (32.6)		
Marital status (n,%)				1.014	0.602
Unmarried	87 (16.4)	41 (15.7)	46 (17)		
Married	422 (79.5)	207 (79.3)	215 (79.6)		
Widowed or Divorced	22 (4.1)	13 (5)	9 (3.3)		
Smokers (n,%)				0.193	0.661
No	498 (93.8)	246 (94.3)	252 (93.3)		
Yes	33 (6.2)	15 (5.7)	18 (6.7)		
Regular diet (n,%)				8.971	0.003
No	73 (13.7)	24 (9.2)	49 (18.1)		
Yes	458 (86.3)	237 (90.8)	221 (81.9)		
Balanced diet (n,%)				13.575	<0.001
No	108 (20.3)	36 (13.8)	72 (26.7)		
Yes	423 (79.7)	225 (86.2)	198 (73.3)		
Number of pregnancies (n,%)				6.245	0.629
0	114 (21.5)	57 (21.8)	57 (21.1)		
1	113 (21.3)	54 (20.7)	59 (21.9)		
2	132 (24.9)	68 (26.1)	64 (23.7)		
3	80 (15.1)	39 (14.9)	41 (15.2)		
4	63 (11.9)	25 (9.6)	38 (14.1)		
5	13 (2.4)	8 (3.1)	5 (1.9)		
6	10 (1.9)	5 (1.9)	5 (1.9)		
7	1 (0.2)	1 (0.4)	0		
10	5 (0.9)	4 (1.5)	1 (0.4)		
Childbirth (n,%)				8.255	0.093
0	143 (26.9)	67 (25.7)	76 (28.1)		
1	237 (44.6)	125 (47.9)	112 (41.5)		
2	126 (23.7)	53 (20.3)	73 (27)		
3	23 (4.3)	15 (5.7)	8 (3)		
4	1 (0.2)	0	1 (0.4)		
5	1 (0.2)	1 (0.4)	0		
Abortion (n,%)				19.053	0.004
0	254 (47.8)	132 (50.6)	122 (45.2)		
1	154 (29)	73 (28)	81 (30)		
2	75 (14.1)	29 (11.1)	46 (17)		
3	27 (5.1)	15 (5.7)	12 (4.4)		
4	10 (1.9)	2 (0.8)	8 (3)		
5	6 (1.1)	6 (2.3)	0		
6	1 (0.2)	0	1 (0.4)		
7	4 (0.8)	4 (1.5)	0		
Cervical ectropion (n,%)				57.708	<0.001
No	263 (49.5)	168 (64.4)	95 (35.2)		
Yes	198 (37.3)	56 (21.5)	142 (52.6)		
Unclear	70 (13.2)	37 (14.2)	33 (12.2)		
Vulva cleaning (n,%)				23.747	<0.001
Clean myself	146 (27.5)	48 (18.4)	98 (36.3)		
Husband cleaning	0	0	0		
Both clean	352 (66.3)	199 (76.2)	153 (56.7)		
Neither clean	33 (6.2)	14 (5.4)	19 (7)		
Method of contraception (n,%)				22.022	<0.001
Contraceptive	27 (5.1)	6 (2.3)	21 (7.8)		
Condom	319 (60.1)	169 (64.8)	150 (55.6)		
intrauterine	91 (17.1)	34 (13)	57 (21.1)		
ligation	25 (4.7)	19 (7.3)	6 (2.2)		
uncontraception	69 (13)	33 (12.6)	36 (13.3)		
**Sleep schedule (mean,se)**	7.07 ± 1.09	7.18 ± 0.98	6.96 ± 1.18	-2.537	0.011
**Age of first sexual intercourse (mean,se)**	21.99 ± 2.9	22.18 ± 2.99	21.81 ± 2.78	-2.392	0.017
**Sexual partners (mean,se)**	1.56 ± 1.05	1.31 ± 0.69	1.78 ± 1.26	-5.554	<0.001

### 3.3 Univariate analysis of study populations based on HPV infection

Significant differences were observed between the HPV-positive and HPV-negative groups across several factors, including occupation, family income, cervical columnar epithelial ectopia, vulvar hygiene practices, contraceptive methods, dietary habits, balanced diet, age at first sexual intercourse, number of previous sexual partners, sleep duration, number of miscarriages, and the number of infections with Chlamydia, gonococcus, Trichomonas vaginalis, and mycobacteria (p<0.05) (**Table 2**).

**Table 2:** Univariate analysis of study populations based on HPV infection.

Characteristic	Total		
	OR	95%CI	P
Profession			
Freelance or housewife			<0.001
Manual labor or services	1.934	1.199-3.118	0.007
Institutional or specialized technical staff	0.803	0.477-1.35	0.407
Others	1.963	1.245-3.096	0.004
household income			
≤3000			0.004
3000-5999	2.681	1.47-4.891	0.001
6000-8999	1.41	0.761-2.612	0.275
≥9000	1.521	0.847-2.733	0.16
Cervical ectropion			
No			<0.001
Yes	4.484	3.01-6.681	<0.001
Unclear	1.577	0.926-2.686	0.093
Vulva cleaning			
Clean myself			<0.001
Husband cleaning			
Both clean	0.377	0.251-0.564	<0.001
Neither clean	0.665	0.307-1.438	0.3
Method of contraception			
Contraceptive			<0.001
Condom	0.254	0.1-0.645	0.004
intrauterine	0.479	0.176-1.304	0.15
ligation	0.09	0.025-0.328	<0.001
uncontraception	0.312	0.112-0.867	0.026
**Regular diet**	0.457	0.271-0.769	0.003
**Balanced diet**	0.44	0.282-0.685	<0.001
**Age of first sexual intercourse**	0.947	0.868-1.034-1.034	0.146
**Sexual partners**	1.759	1.396-2.216	<0.001
**Chlamydia count**	2.011	1.29-3.135	0.002
**Gonococcal count**	14.219	1.856-108.923	0.011
**vaginal trichomonas count**	1.365	1.036-1.797	0.027
**mycotic infection**	1.53	1.225-1.912	<0.001
**Sleep schedule**	0.826	0.704-0.969	0.019
Abortion			
0			0.378
1	1.201	0.804-1.792	0.371
2	1.716	1.014-2.904	0.044
3	0.866	0.39-1.923	0.723
4	4.328	0.901-20.78	0.067
5	0	0	0.999
6	0	0	1
7	0	0	0.999

CI, confidence interval; OR, odds ratio.

### 3.4 Binary logistic regression analysis based on HPV infection

Variables with p<0.05 in the univariate analysis were included in a binary logistic regression model, with factors such as occupation, household income, cervical columnar epithelial ectopia, vulvar hygiene practices, contraception, number of previous sexual partners, and number of abortions used to construct the multifactorial logistic regression equations. The results indicated that manual laborers or service workers (OR=2.138, 95% CI 1.19-3.842, P=0.011) and individuals in other occupations (OR=1.816, 95% CI 1.036-3.184, P=0.037) had a higher risk of HPV infection compared to freelancers or housewives. A household income of 3000-5999 (OR=2.634, 95% CI 1.266-5.481, P=0.01) was associated with a higher risk of HPV infection compared to an income of ≤3000. Patients with cervical columnar epithelial ectopia (OR=4.356, 95% CI 2.754-6.892, P<0.001) had a significantly higher risk of HPV infection compared to those without the condition. In terms of vulvar hygiene, washing with assistance (OR=0.396, 95% CI 0.244-0.642, P<0.001) was associated with a lower risk of HPV infection compared to self-washing. Among contraceptive methods, condom use (OR=0.241, 95% CI 0.062-0.94, P=0.04) and tubal ligation (OR=0.102, 95% CI 0.017-0.596, P<0.001) were linked to a lower risk of HPV infection compared to using oral contraceptives. The number of previous sexual partners was also identified as a risk factor, with a higher number of partners correlating with an increased risk of HPV infection (**Table 3**).

**Table 3:** Binary logistic regression analysis based on HPV infection.

variable	OR,95%CI,P-Value		
	Total	non- menopausal	Upper middle- income groups
Profession			
Freelance or housewife	1	1	1
Manual labor or services	2.138 (1.19-3.842) 0.011	2.205 (1.132-4.295)0.02	2.313 (1.219-4.389)0.01
Institutional or specialized technical staff	0.855 (0.446-1.638)0.636	0.913 (0.445-1.876)0.805	0.851 (0.42-1.722)0.654
Others	1.816 (1.036-3.184)0.037	1.925 (0.999-3.709)0.05	2.259 (1.206-4.234)0.011
household income			
≤3000	1	1	
3000-5999	2.634 (1.266-5.481)0.01	2.115 (0.868-5.158)0.099	1
6000-8999	1.391 (0.649-2.985)0.396	1.08 (0.433-2.695)0.869	0.539 (0.303-0.962)0.036
≥9000	1.11 (0.533-2.311)0.781	0.874 (0.36-2.121)0.767	0.406 (0.228-0.725)0.002
Cervical ectropion			
No	1	1	1
Yes	4.356 (2.754-6.892)<0.001	5.168 (3.076-8.682)<0.001	5.494 (3.308-9.123)<0.001
Unclear	1.305 (0.709-2.404)0.392	1.644 (0.815-3.317)0.165	1.272 (0.657-2.46)0.475
Vulva cleaning			
Clean myself	1	1	1
Husband cleaning			
Both clean	0.396 (0.244-0.642)<0.001	0.509 (0.297-0.872)0.014	0.432 (0.256-0.729)0.002
Neither clean	0.603 (0.239-1.522)0.284	0.278 (0.076-1.017)0.053	1.06 (0.349-3.216)0.919
Method of contraception			
Contraceptive	1	1	1
Condom	0.241 (0.062-0.94)0.04	0.132 (0.024-0.713)0.019	0.113 (0.014-0.946)0.044
intrauterine	0.314 (0.076-1.302)0.11	0.093 (0.016-0.548)0.009	0.178 (0.02-1.582)0.122
ligation	0.102 (0.017-0.596)0.011	0.032 (0.003-0.308)0.003	0.048 (0.004-0.592)0.018
uncontraception	0.298 (0.071-1.248)0.097	0.134 (0.022-0.807)0.028	0.116 (0.013-1.037)0.054
**Sexual partners**	1.75 (1.336-2.292)<0.001	1.652 (1.237-2.206)0.001	1.722 (1.301-2.279)<0.001

CI, confidence interval; OR, odds ratio.

### 3.5 Clinical characteristics and prevalence of HPV and STIs

The statistical analysis revealed significant differences in the rates of Chlamydia, gonococcus, and mycobacterial infections between the HPV-positive and HPV-negative groups, with p values less than 0.05 indicating statistical significance. Additionally, the prevalence of lower genital tract infections was significantly higher among those testing positive for HPV compared to those without the virus. Among the HPV-positive individuals, mycobacterial infections were the most common, accounting for 38.9% (105/270), followed by Trichomonas vaginalis infections at 19.3% (52/270), Chlamydia infections at 13.3% (36/270), and gonococcus infections at 5.2% (14/270) (**Table 4**).

**Table 4:** Prevalence of Reproductive tract infections in HPV-infected and HPV-uninfected populations.

Reproductive tract infections	Total	HPV		χ2-value	P-value
		negetive	positive		
Chlamydia				11.511	0.001
No	484 (91.1)	249 (95.4)	235 (87)		
Yes	47 (8.9)	12 (4.6)	35 (13)		
gonococcus				11.148	0.001
No	516 (97,2)	260 (99.6)	256 (94.8)		
Yes	15 (2.8)	1 (0.4)	14 (5.2)		
trichomonas vaginalis				3.798	0.051
No	445 (83.8)	227 (87.0)	218 (80.7)		
Yes	86 (16.2)	34 (13.0)	52 (19.3)		
mycotic infection				14.871	<0.001
No	365 (68.7)	200 (76.6)	165 (61.1)		
Yes	166 (31.3)	61 (23.4)	105 (38.9)		

### 3.6 Anxiety analysis of the study population

The analysis revealed a significant difference in anxiety levels between the HPV-positive and HPV-negative groups (p < 0.01; **Table 5**). A detailed examination of anxiety among the HPV-positive cohorts showed significant differences in anxiety levels (p < 0.05; **Table 6**). Specifically, individuals positive for HPV types 16 and 18 (Group 1 + Group 2) displayed significantly higher anxiety compared to those positive for other high-risk HPV types (Group 3 + Group 4) across all anxiety grades (p < 0.05; **Table 7**). Moreover, a significant difference was observed between HPV-positive groups with cytologically normal results (Group 1 + Group 3) and those with abnormal cytological findings (Group 2 + Group 4) across all four anxiety levels (p < 0.05; **Table 7**). Logistic regression analysis (Model I) found that HPV positivity (OR=33.837, 95% CI [1.92, 7.67], P < 0.005) was a significant risk factor for anxiety. After adjusting for confounding factors such as the number of abortions, dietary patterns, and Chlamydia infection, the effect of HPV positivity on anxiety remained statistically significant in Model II (OR=2.369, 95% CI [1.134, 4.948], P = 0.022). HPV positivity was thus identified as an independent risk factor for anxiety (**Table 8**).

**Table 5:** The effect of HPV-positive versus HPV-negative status on anxiety.

HPV	Total	Anxiety Rating				P-value
		normal	mild anxiety	moderate anxiety	high anxiety	
HPV (-)	261 (49.2)	250 (51.8)	8 (25.8)	2 (18.2)	1 (16.7)	0.002
HPV (+)	270 (50.8)	233 (48.2)	23 (74.2)	9 (81.8)	5 (83.3)	

**Table 6:** Impact of HPV-positive infections on anxiety in 4 groups.

research group	Total	Anxiety Rating				P-value
		normal	mild anxiety	moderate anxiety	high anxiety	
group1	55 (23)	49 (23.8)	5 (23.8)	1 (14.3)	0	0.002
group2	33 (13.8)	21 (10.2)	7 (33.3)	4 (57.1)	1 (20)	
group3	89 (37.2)	82 (39.8)	4 (19)	2 (28.6)	1 (20)	
group4	62 (25.9)	54 (26.2)	5 (23.8)	0	3 (60)	

group1, HPV 16/18 positive, normal cytology; group2, HPV 16/18 positive, abnormal cytology; group3, Other high-risk hpv positive, normal cytology; group4, Other high risk hpv positive, abnormal cytology.

**Table 7:** Correlation analysis of different study groups with anxiety.

clusters	Total	Anxiety Rating				P
		normal	mild anxiety	moderate anxiety	high anxiety	
HPV 16/18 (+)	88 (36.8)	70 (34)	12 (57.1)	5 (71.4)	1 (20)	0.034
Other hr-hpv (+)	151 (63.2)	136 (66)	9 (42.9)	2 (28.6)	4 (80)	
TCT normal	144 (60.3)	131 (63.6)	9 (42.9)	3 (42.9)	1 (20)	0.049
TCT abnormal	95 (39.7)	75 (36.4)	12 (57.1)	4 (57.1)	4 (80)	

group 1, HPV 16/18 positive, normal cytology; group 2, HPV 16/18 positive, abnormal cytology; group 3, Other high-risk hpv positive, normal cytology; group 4, Other high risk hpv positive, abnormal cytology.

HPV 16/18 (+), group1+group2, Other hr-hpv (+), group3+group4, TCT normal, group1+group3, TCT abnormal, group2+group4.

**Table 8:** Correlation analysis between anxiety levels and study groups.

exposure	Model I	Model II
	OR,95%CI,p-value	OR,95%CI,p-value
HPV		
HPV(-)	1	1
HPV(+)	3.837 (1.92-7.67) <0.001	2.369 (1.134-4.948) 0.022

Model 1: adjust for none. Model 2: adjust for frequency of abortion, dietary patterns, chlamydia, gonococcal infections, mycobacterial infections. CI, confidence interval; OR, odds ratio.

### 3.7 Comparative analysis of the study groups in terms of sexual function scores

To identify independent risk factors associated with TCT infection in the study group, a binary logistic regression analysis was performed, integrating variables from the one-way ANOVA results. Initially, Model I examined the relationship between sexual function and TCT infection, revealing a negative correlation in HR-HPV-positive patients, with an odds ratio (OR) of 0.96 and a 95% confidence interval (CI) ranging from 0.938 to 0.992, achieving statistical significance (P=0.016). In Model II, which included additional variables such as sexual function, Chlamydia, gonococcus, Trichomonas vaginalis, mycobacterial infection, and cervical columnar epithelial ectopia, the negative association between sexual function and TCT infection in HR-HPV-positive patients was confirmed, with an OR of 0.956 and a 95% CI ranging from 0.924 to 0.99 (P=0.012). These findings suggest that sexual function is negatively associated with TCT abnormalities in HR-HPV populations, as detailed in **Table 9**. Additionally, abnormal TCT results were associated with lower FSFI scores compared to normal TCT results (P<0.05, **Table 10**). Significant differences in FSFI scores were also observed across different cytological grades (P<0.05, **Table 11**).

**Table 9:** An analysis of the correlation between high-risk HPV cytology and sexual function.

exposure	Model I	Model II
	OR,95%CI,p-value	OR,95%CI,p-value
FSFI	0.96 (0.928-0.992) 0.016	0.956 (0.924-0.99) 0.012

Model 1: No other covariates were included; Model 2: Incorporation of Chlamydia, Gonococcus, Trichomonas vaginalis, mycobacterial infections, and cervical columnar epithelial ectasia for model2 construction.

CI, confidence interval; OR, odds ratio.

**Table 10:** Differences between HPV16/18 positivity and other HR-HPV positivity at FSFI.

	HPV 16/18 positive	Other high-risk hpv positive	Z	P
FSFI	18.61 ± 5.78	20.74 ± 8.79	-3.584	<0.001

**Table 11:** Differential profile of FSFI between cytologic results.

	TCT abnormal	TCT normal	Z	P
FSFI	18.43 ± 8.57	20.96 ± 7.23	-2.647	0.008

### 3.8 Sensitivity analysis

All analyses were repeated after excluding the menopausal population, and the associations between occupation, income, cervical columnar epithelial ectopia, vulvar hygiene practices, contraceptive use, and the number of previous sexual partners with HPV infection remained consistent. The associations also did not materially change in the sensitivity analyses that excluded low-income populations (<3000) (**Table 3**). Furthermore, when excluding participants with low-risk HPV infection, the associations remained substantially unchanged (**Table 11**).

## 4 Discussion

The geographical distribution of cervical HPV infections is strongly influenced by various demographic factors, including ethnicity, overall health, socioeconomic status, and education levels. These factors play a critical role in the differing prevalence of HPV across regions. A comprehensive study conducted across three distinct centers, which included both rural and urban areas and regions with varying economic conditions, reported that 45% of individuals tested positive for high-risk HPV (HR-HPV) types. The study also revealed substantial regional differences in the prevalence of specific HPV subtypes. In Shenzhen, the predominant subtypes identified were HPV52, HPV18, and HPV16, while in Shaanxi, the most frequently detected subtypes were HPV52, HPV18, and HPV58. In the Northeastern region, HPV52, HPV16, and HPV18 were the leading subtypes. These findings align with previous research that identified regional disparities in HPV subtype distribution (15–17). Based on these results, it is recommended that healthcare providers managing HPV-positive patients pay special attention to those infected with more pathogenic HPV subtypes, as informed by local HPV epidemiological data. The ongoing research into the regional differentiation of HPV subtypes remains incomplete and highlights the need for further extensive studies to validate and build upon these initial findings. Such investigations are crucial for improving cervical cancer prevention, screening, and treatment strategies. By developing HPV vaccines tailored to the specific epidemiological profiles of each region, the precision and efficacy of interventions aimed at reducing the incidence of cervical cancer can be significantly improved (18, 19). This targeted approach allows for more effective strategies to combat the diverse patterns of HPV infection prevalent among various demographic groups, potentially leading to better public health outcomes.

Behavioral factors play a critical role in the development of HPV infection and its progression to cervical cancer. In this study, we investigated risk factors for HPV infection, and our findings align with previous research, confirming that manual laborers, service workers, individuals in other occupations, low- and middle-income groups, cervical columnar epithelial ectasia, and an increase in the number of previous sexual partners are associated with a higher risk of HPV infection. The growing focus on identifying risk factors for HPV infection aims to improve prevention efforts against cervical lesions. Numerous studies have demonstrated that lower genital tract infections can significantly increase the persistence of HPV infection (20, 21). In our analysis, we observed a high prevalence of mycobacterial, Trichomonas vaginalis, and chlamydial infections among HPV-infected individuals. Meta-analyses have identified Chlamydia trachomatis as an independent prognostic marker for cervical cancer, with an odds ratio (OR) of 1.76 and a 95% confidence interval (CI) ranging from 1.03 to 3.01 (22). Analysis of its causes: chlamydial infection can damage the epithelial cells of the mucosa of the reproductive tract, causing changes in the local pH value of the cervix, thus inducing the occurrence of local inflammatory reactions, leading to the impairment of the local immune barrier and increasing the rate of HPV infection. Further basic research is needed to explain the specific underlying mechanisms. In light of these findings, there is a strong case for enhancing Chlamydia trachomatis screening protocols, particularly among women at high risk, including those with concurrent HPV infections. The co-presence of HPV significantly increases their risk profile. Expanding screening measures for Chlamydia trachomatis in these high-risk groups could help reduce the incidence of cervical cancer by promoting early detection and treatment of Chlamydia trachomatis infections, thereby addressing a major factor contributing to the development of this malignancy.

The findings of this study indicate that women with a positive HPV diagnosis exhibit significantly higher levels of anxiety compared to those with a negative diagnosis. When analyzing anxiety in relation to different HPV types, it was observed that HPV types 16 and 18 caused notably greater anxiety than other high-risk HPV types. Furthermore, among HR-HPV-positive women, those with abnormal cytological results experienced higher anxiety levels than those with normal cytology. The highest anxiety levels were observed in women who were both positive for HPV types 16/18 and had abnormal cytological findings, consistent with research by Laura et al. (8). Their study tracked anxiety levels at 6 and 12 months, demonstrating that increased anxiety and distress following a positive HPV result decreased within 6 months but remained elevated for up to 12 months. Similar conclusions have been drawn by other studies (12, 23), all of which emphasize the increased psychosocial burden in HPV-positive individuals. The initial anxious reaction may be influenced by how the diagnosis is communicated. Women who test positive for HPV often express a desire to discuss their results privately with a physician to gain more information, and being well-informed is essential for reducing anxiety related to abnormal results. Additionally, a study by Alay et al. (24) investigated the impact of specific HPV genotypes on anxiety and sexual satisfaction. It was found that women diagnosed with HPV types 16 or 18 experienced significantly higher anxiety. The analysis of our study highlights the importance of addressing the psychological impact of HPV diagnosis, particularly in women diagnosed with high-risk types such as HPV 16 and 18. The consistent findings across various studies regarding increased anxiety levels in HPV-positive women highlight the need for comprehensive care that includes psychological support for those undergoing screening and treatment for HPV infections. Future research should continue to explore the psychosocial aspects of HPV diagnosis. An in-depth examination of factors contributing to adverse psychosocial conditions, such as the manner of delivering an HPV diagnosis, providing detailed information about the virus, and explaining the formation and progression of cervical cancer, could help alleviate anxiety in HPV-infected individuals. Identifying ways in which healthcare providers can better support affected individuals will be critical in improving patient outcomes.

Leite et al. (10) demonstrated that a positive HPV test result is often associated with increased psychological distress, particularly anxiety symptoms. Our study similarly found significant variations in FSFI scores among HR-HPV-positive patients. Furthermore, different HR-HPV types had varying effects on FSFI scores, with all subgroups showing a notable decline in sexual function. The reduction in FSFI scores was particularly pronounced in Group 2, indicating that sexual dysfunction was more severe in women who were positive for HPV16/18 and had cytological abnormalities. Several studies (25, 26) have also reported that HPV infection, combined with cytological abnormalities, significantly affects women’s sexual relationships. Kwan et al. suggest that the distress experienced by women with both HPV positivity and cytological abnormalities is exacerbated by several factors, including fear of cancer, awareness of HPV infection, and the anticipation of an upcoming colposcopy (27, 28). Further follow-up revealed that the disruption in sexual relationships was most significant in the early stages of HPV diagnosis. However, as women gained a better understanding of disease transmission, they were able to resume sexual activity safely (12, 24, 29).

A qualitative study (26) revealed that women’s anxiety levels following a positive HPV test result were significantly influenced by their interpersonal habits, social and cultural norms, relationship experiences, and understanding of the HPV virus. Participants expressed various negative emotions, including shame, anger, and fear. Many attributed their positive result to their partner’s infidelity and expressed concerns about the potential progression to cervical cancer. Some women reported that knowledge of their HPV infection made it difficult to form new sexual relationships. McCaffery et al. (26) emphasized that HPV-positive women often experience negative feelings about their sexual behavior, such as fear of disclosing their diagnosis to partners, feelings of guilt or uncleanliness, fear of transmitting the virus, anxiety about potential sexual rejection, and anger towards their partner’s perceived betrayal. These emotional responses can lead to sexual dysfunction, including reduced libido and pain during intercourse. Monsonego et al. (30) conducted a study on the psychological impact and informational needs of women undergoing cervical cancer screening in three European countries. The majority of women reported that after receiving abnormal results, they did not receive sufficient information or psychological support from healthcare providers. Women with HPV emphasized the need for comprehensive information and emotional support throughout the diagnostic process (25). Similarly, Dieng et al. (31) analyzed the informational needs of Australian women regarding cervical screening and found that most women wanted detailed information about the risks and benefits of screening. The study also identified a link between the style of counseling provided and women’s psychological responses to an HPV diagnosis. These findings highlight the importance of pairing HPV testing with thorough health education. Healthcare providers must ensure that women undergoing cervical screening are well-informed and work to reduce the stigma associated with viral infections. This approach is essential for minimizing the social and psychological harms associated with cervical screening.

Research on the impact of cervical cancer screening results on women’s anxiety levels and sexual functioning in China is limited. This study has certain limitations, including the absence of long-term follow-up for women experiencing anxiety, insufficient geographic coverage, and a lack of comprehensiveness in the survey, making it unclear whether anxiety and sexual dysfunction reduce over time. Nevertheless, the findings provide valuable insights that may inform the development of HPV vaccination programs and cervical cancer prevention strategies in the region. The study highlights the importance of healthcare professionals in raising public awareness about HPV. This can be accomplished through educational lectures, counseling services, and the implementation of sustainable intervention plans aimed at improving women’s psychosocial adjustment to HPV testing. These efforts can help reduce the negative impact of HPV on couples’ sexual health.

## 5 Conclusion

In conclusion, this study revealed that women with positive HPV test results exhibited significantly higher anxiety levels compared to their HPV-negative counterparts. HPV types 16 and 18, along with abnormal cytology, were more likely to lead to increased anxiety and sexual dysfunction in women with HPV infection. Furthermore, the research identified a significant relationship between HPV infection and lower genital tract infections. These insights provide a comprehensive understanding of the multifaceted effects of HPV infection and highlight the necessity of integrating psychological support into HPV prevention and treatment strategies. By tailoring information and management strategies to different geographic regions, this holistic approach can improve patient outcomes by addressing both the medical and emotional needs of women undergoing HPV screening and treatment.

## Data Availability

The original contributions presented in the study are included in the article/supplementary material. Further inquiries can be directed to the corresponding author.
